# Supplementary figures and images for: Ventral tegmental area interneurons revisited: GABA and glutamate projection neurons make local synapses
Source: bioRxiv. 2025 Jan 17:2024.06.07.597996. Originally published 2024 Jun 8. Preprint. [Version 2] doi: 10.1101/2024.06.07.597996 (PMC11185768; doi:10.1101/2024.06.07.597996)

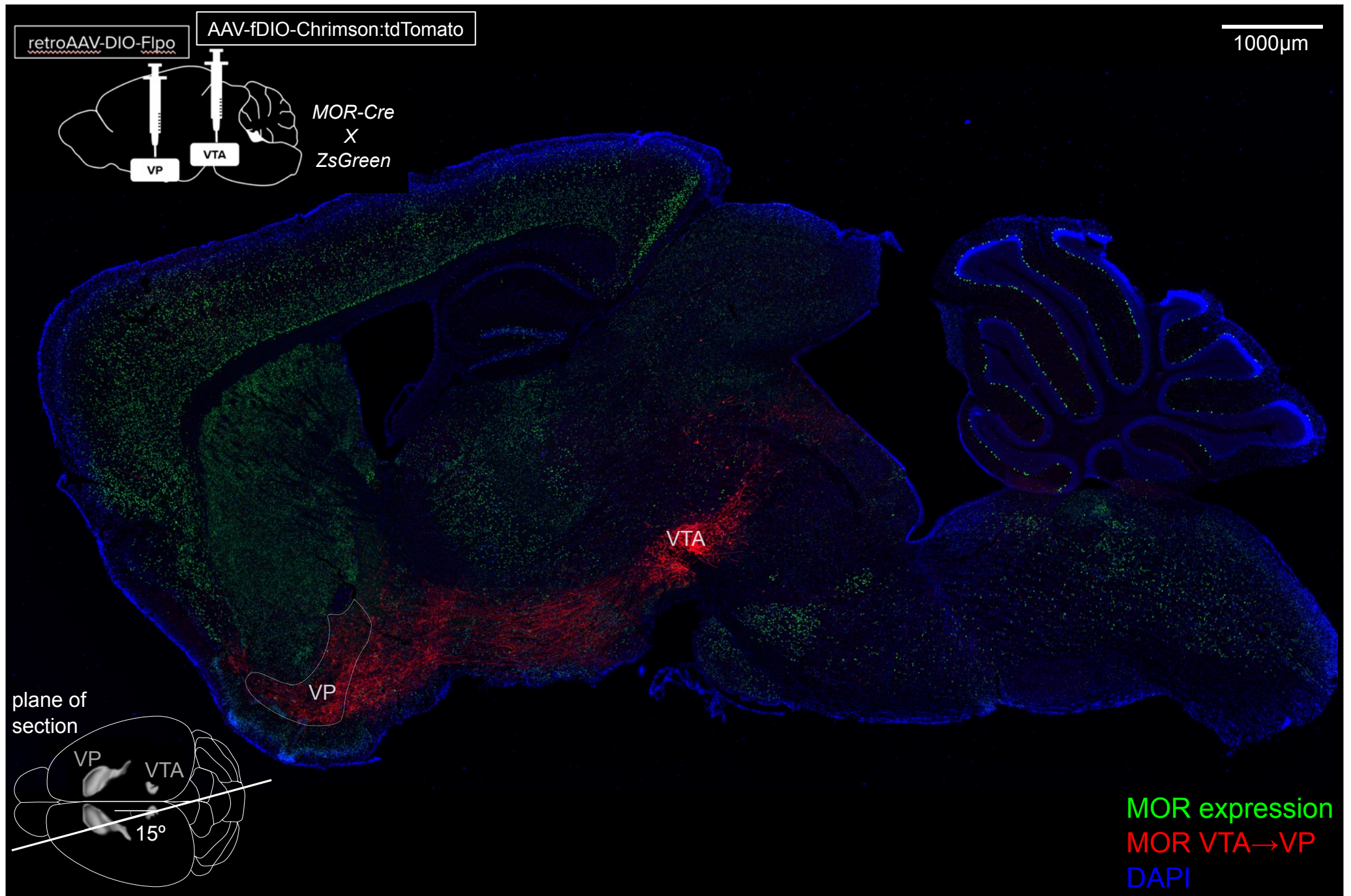

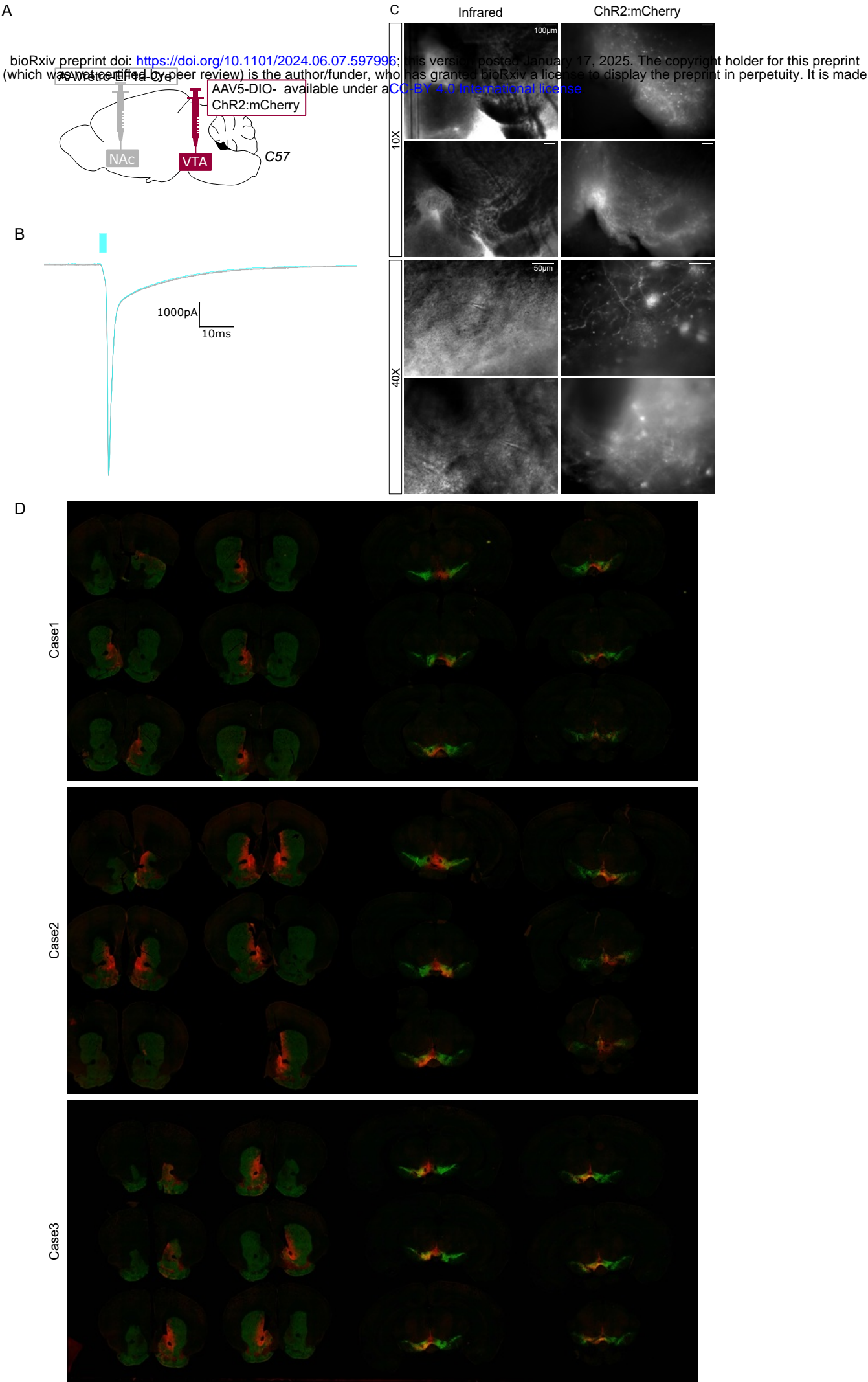

A      Histological cases for ephys prep VTA to VP

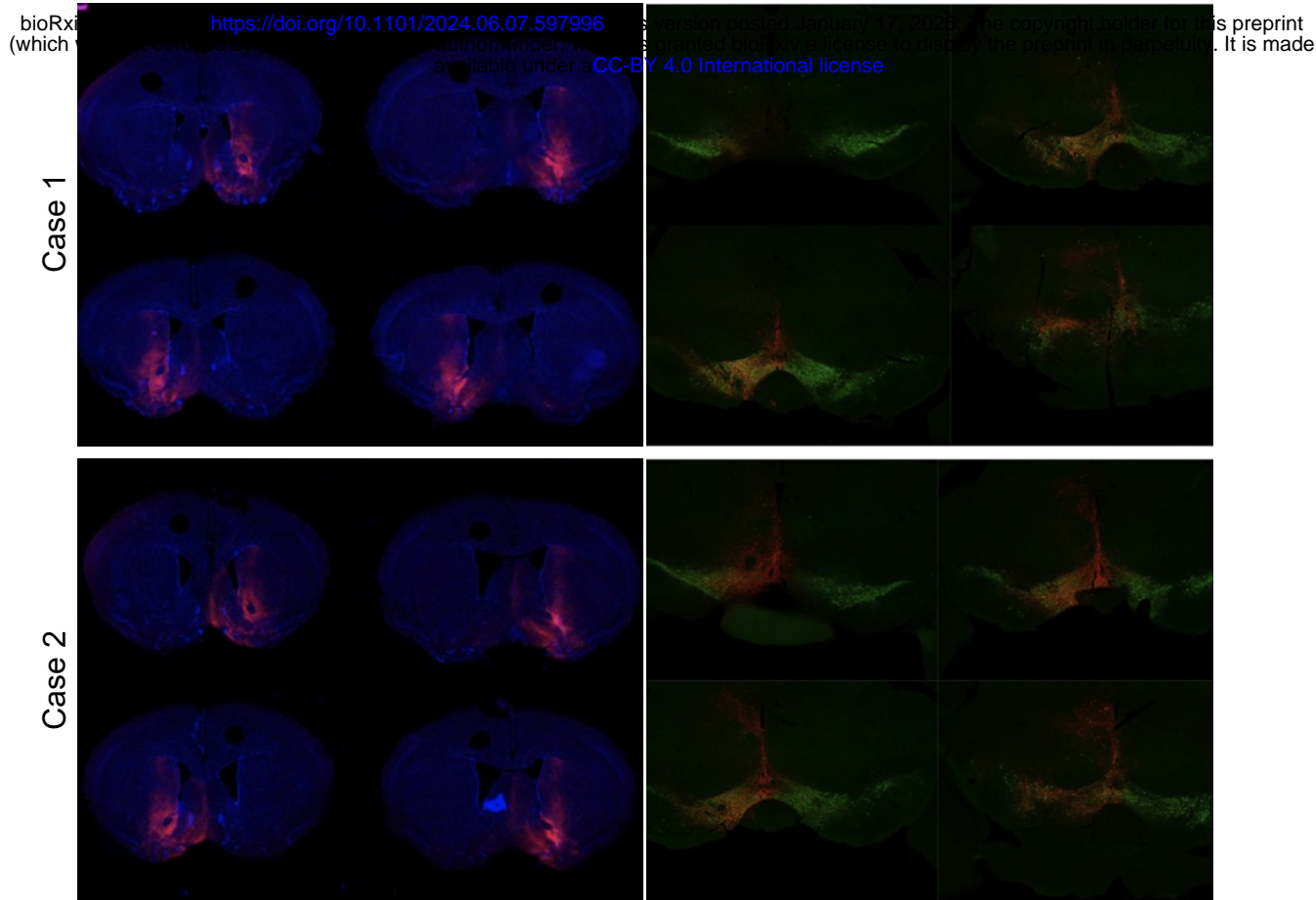

B      Histological cases for ephys prep VTA to PFC

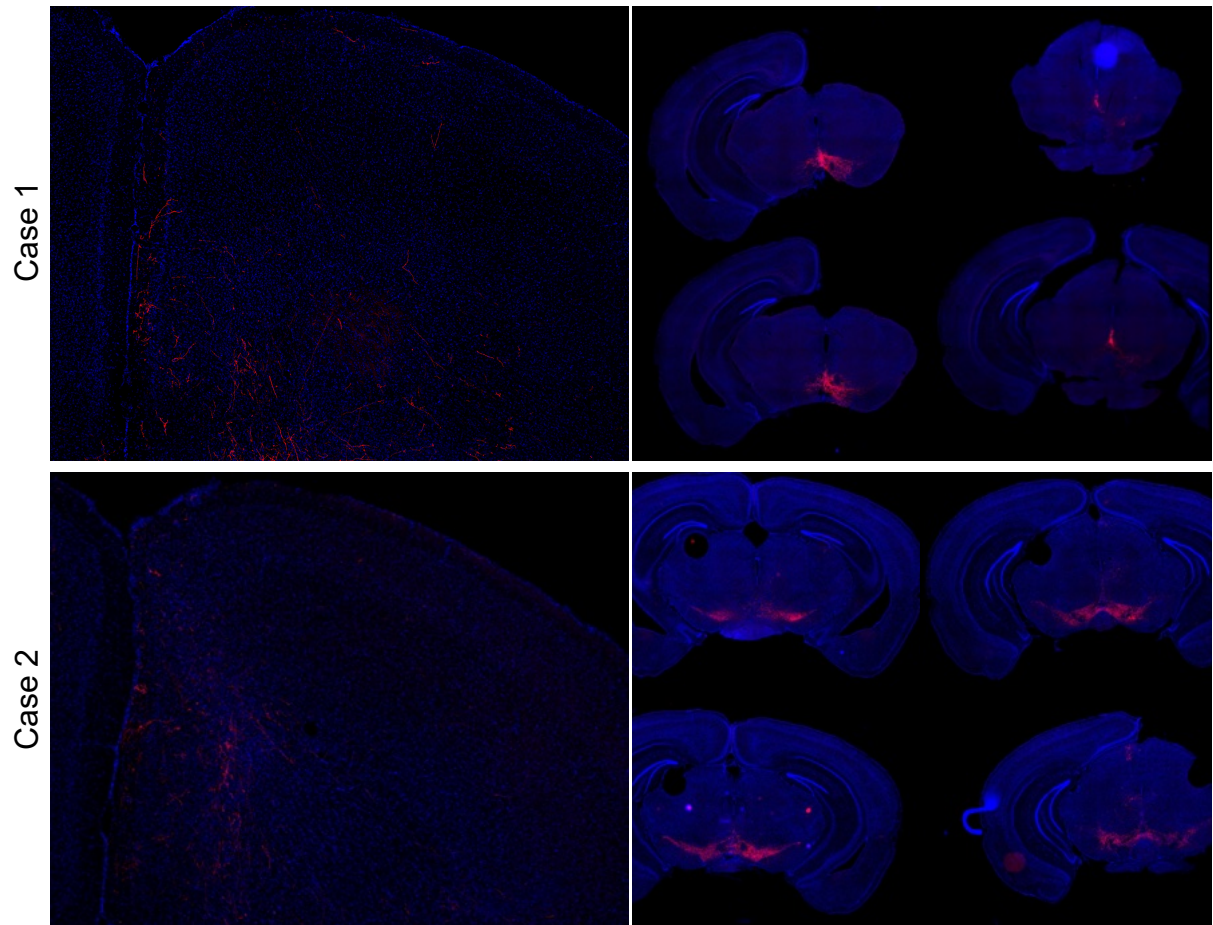

Supplement: 1 — Supplemental Figure 1: Projection of MOR-Cre-expressing VTA neurons to VP (related to Figure 3). Sagittal image, genotypes, and schematics of dual AAV approach and approximate location and sectioning angle of the cut. ZsGreen (green) labels all cells that have expressed MOR-Cre, Chrimson:tdTomato (red) labels cells/fibers from MOR-Cre VTA neurons projecting to VP, DAPI (blue) labels nuclei. Supplemental Figure 2: Photocurrent and histological validation of approach used in Figure 5 (related to Figure 5). (A) Dual AAV approach to express ChR2:mCherry in NAc-projecting VTA neurons in wild-type mice. (B) Example opsin-mediated photocurrent from ChR2:mCherry-positive neuron of VTA. (C) Example images under DIC IR light and mCherry expression around patch-clamp pipettes. (D) Additional cases of histology. Supplemental Figure 3: Histological validation of approach used in Figure 6 (related to Figure 6). (A) Additional cases of histology with expression of ChR2:mCherry in VP-projecting VTA neurons in wild-type mice. (B) Additional cases of histology with expression of ChR2:mCherry in PFC-projecting VTA neurons in wild-type mice. [file NIHPP-2024.06.07.597996V2-supplement-1.pdf]
