# Supplementary material for: Ventral tegmental area interneurons revisited: GABA and glutamate projection neurons make local synapses
Source: bioRxiv. 2024 Jun 8:2024.06.07.597996. Preprint. [Version 1] doi: 10.1101/2024.06.07.597996 (PMC11185768; doi:10.1101/2024.06.07.597996)

bioRxiv preprint doi: https://doi.org/10.1101/2024.06.07.597996; this version posted June 8, 2024. The copyright holder for this preprint (which was not certified by peer review) is the author/funder, who has granted bioRxiv a license to display the preprint in perpetuity. It is made available under aCC-BY 4.0 International license.

Supplemental Figure 5: photocurrent and histological validation of approach used in Figure 5. (**A**) Dual AAV approach to express ChR2:mCherry in NAc-projecting VTA neurons in wild-type mice. (**B**) Example of photocurrent from ChR2:mCherry-positive neuron of VTA. (**C**) Example images under DIC R light and mCherry expression around patch-clamp pipettes. (**D**) Additional cases of histology.

bioRxiv preprint doi: https://doi.org/10.1101/2024.06.07.597996; this version posted June 8, 2024. The copyright holder for this preprint (which was not certified by peer review) is the author/funder, who has granted bioRxiv a license to display the preprint in perpetuity. It is made available under aCC-BY 4.0 International license.

FS5

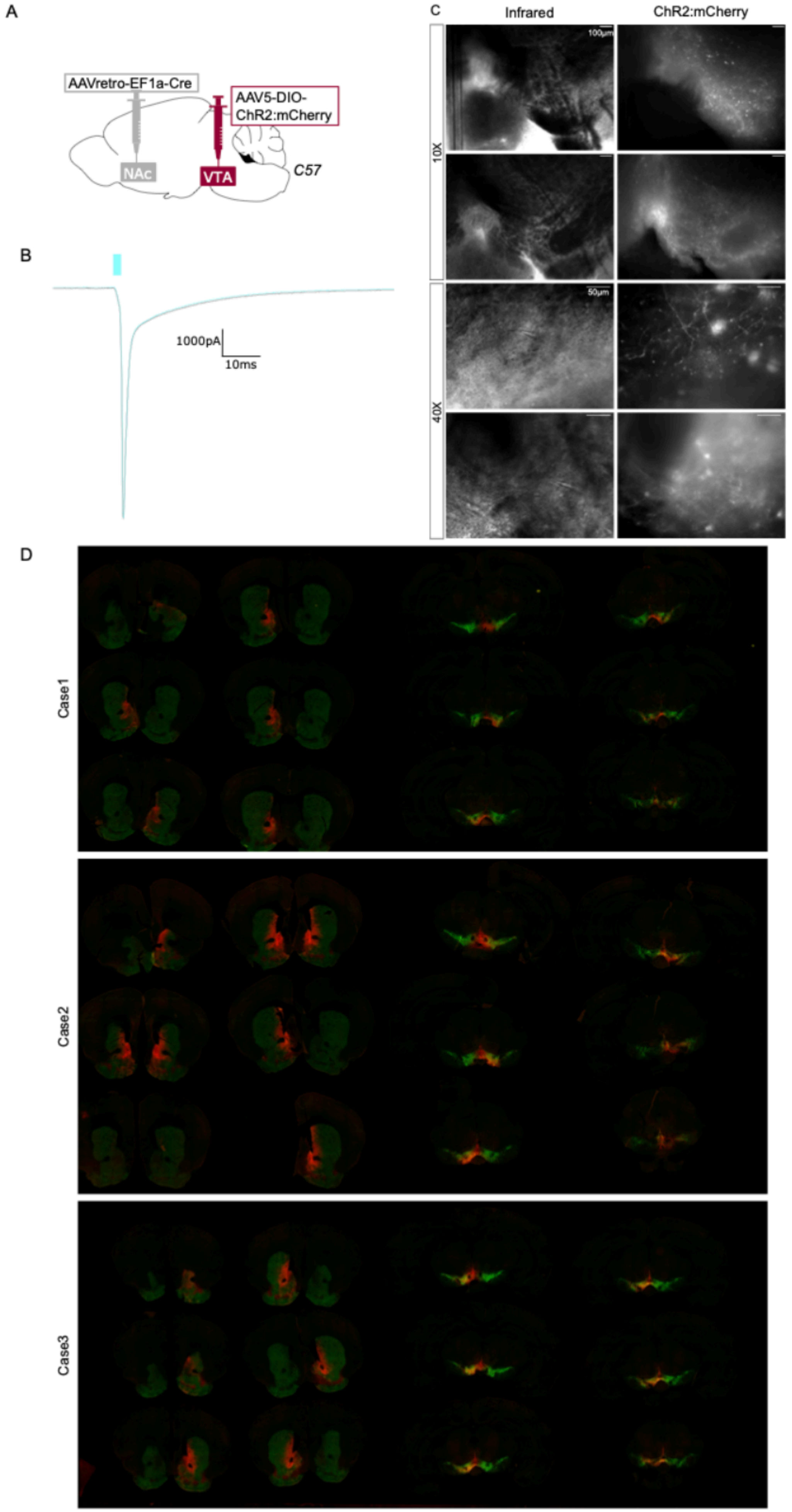

Supplement: Supplementary file 1 [file NIHPP2024.06.07.597996V1-supplement-1.pdf]
